# Epidemiology and viral etiologies of Severe Acute Respiratory Infections (SARI) in the Northern Vietnam

**Published:** 2011-01-10

**Authors:** Nguyen Tran Hien, Nguyen Thi Thuong, Vu Dinh Thiem, Nguyen Quang Minh, Tran Nhu Duong

**Affiliations:** 1 National Institute of Hygiene and Epidemiology, Hanoi, Vietnam

## Background

Severe Acute Respiratory Infections (SARI) is the leading global cause of morbidity and mortality from infectious diseases. Since January 2009, a Sentinel Surveillance System for Severe Acute Respiratory Infections has been established based in a Northern Province of Vietnam (Hai Duong province). The purpose of this program is to describe the epidemiology and identify the viral etiologies of SARI in a province of the North of Vietnam for rapid diagnosis and response to epidemic outbreaks.

## Methods

Patients from a provincial and district hospital of Hai Duong province, Northern Vietnam were enrolled, using standardized case definitions of SARI. Clinical and epidemiological data were collected using structured questionnaire. 18 viral etiologies of SARI were examined from throat swabs of eligible patients using multiplex-polymerase chain reaction (RT-PCR) in the framework of the SISEA project.

## Results

From January 2009 to September 2010, 484 patients were enrolled in the study. The average age of subjects was 12.7 years, ranging from 0 to 90 years and 53.1% were male. Of 484 specimens, 283 (58.47%) were positive for viral agents. Of whom, 218 (77.03%) were mono-infection, 65 (22.97%) had mixed viral infection with at least one other virus. The most common viral etiologies of SARI were seasonal influenza A/H1N1 (16.32%) and rhino virus (15.5%). Pandemic influenza A/H1N1/09 was first detected in this surveillance system in week 42^nd^ of 2009 and lasted for 4 weeks only. After that, there were only 2 cases detected. The proportion of specimens positive with pandemic influenza A/H1N1/09 increased from 50% (week 42^nd^) and became totally predominant (accounted for around 77-100 %) from week 43^rd^ to week 45^th^, and suddenly decreased to 0 % in week 46^th^. Rhinovirus infection seems occurred seasonally with the majority of patients appearing from January to March. However, no seasonality was observed for other specific pathogens causing SARI. Interestingly, this study has detected the presence of a parainfluenza 4 outbreak and of sporadic human FluC cases in 2009. In addition, this study first identified the presence of human corona virus NL63 and HKU1 in Vietnam.

## Conclusions

SARI infections are caused by variety of viruses. The system is sensitive to identify pandemic influenza A/H1N1/09 community transmission. It is necessary to enhance the severe acute respiratory infection surveillance for early detection and rapid response.

